# Paraoxonase Polymorphisms, Haplotypes, and Enzyme Activity in Latino Mothers and Newborns

**DOI:** 10.1289/ehp.8540

**Published:** 2006-02-02

**Authors:** Nina Holland, Clement Furlong, Maria Bastaki, Rebecca Richter, Asa Bradman, Karen Huen, Kenneth Beckman, Brenda Eskenazi

**Affiliations:** 1 Center for Children’s Environmental Health, School of Public Health, University of California, Berkeley, California, USA; 2 Departments of Genome Sciences and Medicine, Division of Medical Genetics, University of Washington, Seattle, Washington, USA; 3 Children’s Hospital Research Institute, Oakland, California, USA

**Keywords:** chlorpyrifos, cord blood, haplotypes, Latino cohort, linkage disequilibrium, organophosphate, paraoxonase 1 (PON1) genotype, paraoxonase activity, pesticides, *PON1* polymorphisms, pregnancy

## Abstract

Recent studies have demonstrated widespread pesticide exposures in pregnant women and in children. Plasma paraoxonase 1 (PON1) plays an important role in detoxification of various organophosphates. The goals of this study were to examine in the Center for Health Assessment of Mothers and Children of Salinas (CHAMACOS) birth cohort of Latina mothers and their newborns living in the Salinas Valley, California, the frequencies of five *PON1* polymorphisms in the coding region (*192_QR_* and *55_LM_*) and the promoter region (−*162_AG_*, −*909_CG_*, and −*108_CT_*) and to determine their associations with PON1 plasma levels [phenylacetate arylesterase (AREase)] and enzyme activities of paraoxonase (POase) and chlorpyrifos oxonase (CPOase). Additionally, we report results of *PON1* linkage analysis and estimate the predictive value of haplotypes for PON1 plasma levels. We found that *PON1*_−_*_909_*, *PON1*_−_*_108_*, and *PON1_192_* had an equal frequency (0.5) of both alleles, whereas *PON1*_−_*_162_* and *PON1_55_* had lower variant allele frequencies (0.2). Nearly complete linkage disequilibrium was observed among coding and promoter polymorphisms (*p* < 0.001), except *PON1_192_* and *PON1*_−_*_162_* (*p* > 0.4). Children’s PON1 plasma levels (AREase ranged from 4.3 to 110.7 U/mL) were 4-fold lower than their mothers’ (19.8 to 281.4 U/mL). POase and CPOase activities were approximately 3-fold lower in newborns than in mothers. The genetic contribution to PON1 enzyme variability was higher in newborns (*R*^2^ = 25.1% by genotype and 26.3% by haplotype) than in mothers (*R*^2^ = 8.1 and 8.8%, respectively). However, haplotypes and genotypes were comparable in predicting PON1 plasma levels in mothers and newborns. Most of the newborn children and some pregnant women in this Latino cohort may have elevated susceptibility to organophosphate toxicity because of their *PON1_192_* genotype and low PON1 plasma levels.

Organophosphate (OP) pesticide exposure remains widespread in the United States (Barr et al. 2004; Bradman et al. 2005; Hill et al. 1995; Loewenherz et al. 1997; Simcox et al. 1999). Pregnant women, fetuses, and children in both urban (Berkowitz et al. 2003; Whyatt et al. 2003) and rural agricultural populations (Eskenazi et al. 2004; Fenske et al. 2002) are directly exposed to pesticides, and in some cases these exposures may exceed health-based reference doses (Bradman et al. 2005; Castorina et al. 2003). OP pesticide metabolites have also been detected in meconium (Whyatt and Barr 2001) and amniotic fluid (Bradman et al. 2003). OP exposure at high doses has profound effects, primarily on the central nervous system (Eskenazi et al. 1999), and there is growing information in animals and humans suggesting that low-level chronic exposure may affect neurodevelopment (Eskenazi et al. 1999; Young et al. 2005).

The unique physiologic and behavioral characteristics of children may increase their exposures to environmental contaminants compared with adults (National Research Council 1993). Young children eat, drink, and breathe more per unit of body weight than do adults, and they also explore their environment orally, engaging in extensive hand-to-mouth behavior (National Research Council 1993). In addition, young children may be more susceptible to the adverse effects of OP exposure than are adults, because of their lower ability to metabolize and detoxify OP pesticides (Padilla et al. 2000; Sheets 2000).

The human paraoxonase 1 (PON1) enzyme (43 kDa, composed of 354 amino acids) is a polymorphic, high-density lipoprotein-associated esterase that metabolizes many different substrates, including OP compounds (Davies et al. 1996; Geldmachervon Mallinckrodt and Diepgen 1988), drugs, and oxidized lipids (Draganov et al. 2005; Watson et al. 1995). Studies of the PON1 enzyme, which detoxifies activated oxon forms of several OP pesticides, including diazinon, chlorpyrifos, and parathion, indicate that PON1 levels in newborns are on average 3- to 4-fold lower than those of adults (Augustinsson and Barr 1963; Chen et al. 2003; Cole et al. 2003; Ecobichon and Stephens 1973; Mueller et al. 1983). Newborns reach a plateau near adult PON1 levels between 6 and 24 months of age, suggesting that newborn children and infants will be more susceptible to OP compounds (Cole et al. 2003).

The *PON1* gene has been mapped to chromosome 7q21.3–22.1 (Humbert et al. 1993; Primo-Parmo et al. 1996) and contains nine exons. Recent studies suggest that some individuals may have specific *PON1* genotypes that are associated with low levels of plasma *PON1* (Brophy et al. 2001b; Deakin et al. 2003; Suehiro et al. 2000). The hydrolytic catalytic efficiency of some *PON1* substrates is dependent on the single nucleotide polymorphism (SNP) *Q192R* (Li et al. 2000). However, adults with the same *PON1_192_* genotype can have at least a 13-fold difference in *PON1* activities (Davies et al. 1996; Furlong et al. 2002). The *C-108T* polymorphism, in a Sp1 binding site of the promoter region, has a major effect on the expression of the *PON1* gene. The *C-108* allele expresses on average twice as much *PON1* as does the *T-108* allele (Brophy et al. 2001b; James et al. 2000). Other polymorphisms in the promoter region (*A-162G*, and *C-909G*) may have less significant effects on PON1 expression and are in strong disequilibrium with *C-108T* (Costa et al. 2002; James et al. 2000). The *PON1_M55_* allele has been associated with low *PON1* enzyme levels; however, most of this effect is related to its strong disequilibrium with the *T-108* allele. Recently, additional promoter polymorphisms have been identified (SeattleSNPs 2005); however, their influence on PON1 levels has yet to be determined (Jarvik GP, personal communication). Limited information on *PON1* haplotypes (Chen et al. 2005; Koda et al. 2004; Wetmur et al. 2005) suggests that haplotypes provide no significant improvement in predicting PON1 levels over a combination of *PON1* polymorphisms (Chen et al. 2005).

The gene frequencies for specific alleles of *PON1* genes vary by ethnicity, implying differential susceptibility to pesticides among different ethnic groups (Allebrandt et al. 2002; Brophy et al. 2002). In a study of mothers and newborns from New York, a noticeable difference in haplotype frequency was observed among three ethnic groups (Chen et al. 2005).

In the present study, we examined the frequencies and haplotypes of five *PON1* polymorphisms in coding regions (*192_QR_* and *55_LM_*) and promoter regions (−*162_AG_*, −*909_CG_*, and −*108_CT_*) and their associations with PON1 plasma levels and enzyme activities in pregnant Latina women and their newborns living in the Salinas Valley, California, an agricultural community (Eskenazi et al. 2003) where approximately 500,000 pounds of OP pesticides are used annually (California Environmental Protection Agency 2002). Additionally, we report results of *PON1* linkage analysis for five *PON1* polymorphisms and estimate the predictive value of haplotypes, compared with *PON1* genotypes, for PON1 plasma levels. The present study follows our recent publications demonstrating that the Salinas Valley population has a relatively high level of exposure to OP compounds (Bradman et al. 2005) and that OP exposure as assessed by maternal dialkyl phosphate metabolite levels was associated with shorter gestational age (Eskenazi et al. 2004) and increased frequency of abnormal reflexes in neonates (Young et al. 2005).

## Materials and Methods

### Subjects and recruitment

Pregnant women (*n* = 130) and their newborns (*n* = 130) were randomly selected from the CHAMACOS (Center for the Health Assessment of Mothers and Children of Salinas) cohort, a longitudinal birth cohort study of the effects of pesticides and other environmental exposures on the health of pregnant women and their children living in the Salinas Valley, California. Women were eligible for enrollment in the CHAMA-COS study if they were ≥ 18 years of age, < 20 weeks’ gestation at enrollment, English- or Spanish-speaking, Medi-Cal eligible, and planning to deliver at the Natividad Medical Center (Bradman et al. 2005; Eskenazi et al. 2003, 2004; Young et al. 2005). All women in the subcohort described here were representative of the CHAMACOS cohort; they were Latina by ethnicity, including 85% born in Mexico and the remainder in the United States. Most of the participants never smoked (> 92%), had relatively high pesticide exposures based on diethyl phosphate urinary metabolites (median, 20 nmol/L; range, 7–560 nmol/L), and worked in agriculture during pregnancy (39%). Fathers were more likely to smoke (11%) and work in agriculture (72%) than were mothers. Study protocols were approved by the University of California, Berkeley, and the University of Washington human-subject review committees in compliance with all applicable requirements. Written informed consent was provided by all subjects.

### Biologic samples collection and processing

We collected blood from mothers at the time of their glucose tolerance test (26.1 ± 2.3 weeks) and in the hospital shortly before or after delivery. Blood samples were also collected from the umbilical cords by delivery room staff once the baby was safely delivered. Heparinized whole blood was centrifuged, divided into plasma, buffy coats, and red blood cells, and then stored at −80°C. BD Vacutainers (Becton Dickinson, Franklin Lakes, NJ) without anticoagulant were used to collect serum and clot. Processed plasma samples were stored at −80°C before being shipped on dry ice to the University of Washington, Seattle, for analysis of enzyme activity.

DNA was isolated from blood clots. Blood clots thawed in a 37°C water bath were first mechanically disrupted using ClotSpin tubes (Gentra Systems Inc., Minneapolis, MN). The Qiagen protocol (Qiagen Inc., Santa Clarita, CA) was slightly modified by prolonging the initial lysis and protease digestion step to overnight incubation. DNA concentration was measured using PicoGreen (Molecular Probes Inc., Eugene, OR), adjusted to 10 ng/μL, plated in 96-well plates, and stored at −80°C. Samples were transferred to 384-well plates for analysis of multiple SNPs, using robotic equipment to avoid manual pipetting errors and for time efficiency.

### PON1 *genotyping*

Genotyping was conducted by the University of California, Berkeley, and Children’s Hospital Research Institute Genotyping Core. Taqman real-time polymerase chain reaction method was used for genotyping of the −*162_AG_*, *55_LM_*, and *192_QR_* polymorphisms. Briefly, primers for these SNPs were custom designed by Applied Biosystems Inc. (Foster City, CA). Amplifluor allele-specific primers were used for genotyping of −*909_CG_* and −*108_CT_*. Genotype calling was performed either manually using a spreadsheet (Chemicon AssayAuditor, for real-time data) or by automatic allele calling in SDS 2.1 (Applied Biosystems, for end-point data).

Quality assurance procedures included assessment of randomly distributed blank samples in each plate, duplicates of randomly selected samples with independently isolated DNA from the same subjects, and internal controls. Repeated analysis in several runs showed a high degree (96.5%) of concordance, and the most robust call was selected in the case of discordance (3.5%). Furthermore, the assays were repeated for all low-confidence samples until a reliable call was obtained, using a combination of the TaqMan and Amplifluor methods for a subset of samples. Additional analysis was performed independently at the University of Washington for 10% of the DNA samples for the *192_QR_* polymorphism by standard polymerase chain reaction method (details given by Richter et al. 2004) with approximately 95% concordance; all discrepancies were resolved by repeated runs. Quality control software was used to check data for Mendelian errors, and if those were noted, the whole run was repeated.

### Enzyme assays

Plasma was frozen at −80°C until analysis. We measured three PON1 enzyme activities in plasma from mothers and children, using paraoxonase (POase), chlorpyrifos oxonase (CPOase), and phenylacetate arylesterase (AREase) according to published protocols (Jarvik et al. 2003; Richter and Furlong 1999; Richter et al. 2004). We used PON1 plasma levels (AREase assay) to analyze the genetic effect because, unlike POase and CPOase levels, they are not affected by differential catalytic efficiency primarily controlled by the *PON1_192_* SNP and have been shown to correspond with PON1 levels determined by immunologic methods (Blatter-Garin et al. 1994; Furlong et al. 1993). Together, these three assays provide comprehensive information about PON1 enzyme activities regarding different substrates. Assessment of PON1 activities in mothers was first conducted for 25 pregnant women at two different time points, at 26 weeks and at delivery. PON1 activities were not statistically different between the two time points for all three PON1 enzyme assays (*r* = 0.77–1.0, *p* < 0.0001). Therefore, we performed analyses of AREase, POase, and CPOase in the remainder of 105 Latina mothers at one time point only—at 26 weeks’ gestation. Children in the study were of both sexes, and girls represented 54%. No sex differences in PON1 enzyme levels or genotypes were observed, as is consistent with the available PON1 literature (Costa et al. 2002).

### Statistical analysis

Standard analyses for all genotype data included analysis for Hardy-Weinberg equilibrium, pairwise linkage disequilibrium (LD), and haplotype assignment using algorithms implemented in the publicly available Haploview software (Battett et al. 2005), including PYPOP (Lancaster et al. 2003), tagSNPs (Stram et al. 2003), and PHASE (Stephens et al. 2001, 2003). The LD statistic *D*′ was calculated for each pair of five *PON1* SNPs, and *R*^2^ values were used to describe the haplotype structure of the *PON1* gene in our Latino cohort. PYPOP, tagSNPs, and PHASE software methods showed similar results. We used PHASE to generate the data reported in this article, because it has been shown to reduce error rates in haplotype reconstruction compared with the expectation maximization algorithm (Stephens and Donelly 2003).

Subjects were grouped according to their imputed diplotypes (Chen et al. 2005). When more than one diplotype was possible for an individual, only the most likely imputed haplotypes were used in this analysis. The distributions and descriptive statistics were established separately for each of the three PON1 enzyme assays in mothers and in their newborns for each of the five SNPs. The distributions of enzyme activities were approximately normal. Linear regression and backward regression models were used to determine whether the additional information for all five polymorphisms altered the effect of genotype on enzyme activity. Coefficients of determination (total *R*^2^) were calculated for the proportion of variability in PON1 plasma levels explained by the five SNP genotypes (used as ordinal variables) and by imputed haplotypes. Each haplotype with > 5% frequency was coded as a variable in the linear regression model, where the values 0, 1, or 2 denoted the presence of zero, one, or two copies of the haplotype for a subject. Haplotypes with < 5% frequency were pooled into one group for this analysis. All analyses were conducted in STATA software (version 8.0; StataCorp., College Station, TX) and SAS software (version 9.1; SAS Institute Inc., Cary, NC).

## Results

### PON1 *polymorphisms*

*PON1* gene frequencies were established for two coding polymorphisms (*PON1_192_* and *PON1_55_*) and three promoter region polymorphisms (*PON1*_−_*_909_*, *PON1*_−_*_162_*, *PON1*_−_*_108_*) (Table 1). As expected, the five polymorphisms had similar allelic frequencies in 130 pregnant Latina women of Mexican descent and their newborns. All genotypes were consistent with Hardy-Weinberg equilibrium (data not shown). The SNPs at position *PON1*_−_*_162_* of the promoter region and *PON1_55_* in the coding region had lower variant allele frequencies (−*162*A, *55*M) than did the major allele, whereas the other three polymorphisms (*PON1*_−_*_909_*, *PON1*_−_*_108_*_,_ and *PON1_192_*) had approximately equal presence of both alleles in this population. Specifically, the frequencies of *PON1_192_* alleles were *Q* = 0.46, *R* = 0.54 in mothers, and *Q* = 0.51, *R* = 0.49 in children, with overall population prevalence Q ~ R ~ 0.5. Frequencies for the major alleles of promoter polymorphisms PON1_G−909_, PON1_G−162_, and PON1_C−108_ were, respectively, 0.52, 0.78, and 0.51 in mothers and 0.56, 0.81, and 0.55 in children, and the frequency of a major allele of the coding *PON1_L55_* polymorphism equaled 0.82 in both age groups.

Results of linkage analysis between five *PON1* polymorphisms were also similar for Latina mothers and their newborns (Table 2). We observed nearly complete LD among the three promoter polymorphisms (*D*′ = 0.8–1; *p* < 0.001). Strong LD (*D*′ = 0.87 and 0.94 in mothers and children, respectively; *p* < 0.001) was found between the two coding polymorphisms (*PON1_192_* and *PON1_55_*). There was a more complex relationship between coding and promoter region polymorphisms: although *PON1_55_* had high LD with all three promoter polymorphisms (*D*′ = 0.74–1), only two (*PON1*_−_*_108_* and *PON1*_−_*_909_*) of the three promoter SNPs were linked to *PON1_192_* (*D*′ = 0.22 and 0.19 in mothers, and *D*′ = 0.27 and 0.34 in children, respectively). These LDs were all modest but statistically significant. However, no linkage was demonstrated between SNPs at positions *PON1_192_* and *PON1*_−_*_162_* (*D*′ = 0.0 in mothers and 0.18 and children; *p* > 0.4).

### Haplotype analysis

Haplotype analysis revealed a total of 32 different combinations of alleles (Table 3). However, their frequencies were noticeably different and fall into three distinct groups: *a*) a main group contributing approximately 93% of all haplotypes for this cohort, which is composed of seven haplotypes with individual frequencies ranging from 7 to 24%; *b*) a second group with individual frequencies ranging from 0.1 to 1.9%, which contributes 5.5–7.8% of all haplotypes in mothers and children; and *c*) a group of 17 rare haplotypes contributing a total of approximately 1% of haplotype variability.

### PON1 *enzyme activities*

AREase levels allow for a comparison of PON1 levels across genotypes because the catalytic efficiency of hydrolysis of phenylacetate is not affected by the *PON1_192_* polymorphism (Tables 4, 5). The AREase activity in mothers ranged from 19.8 to 281.4 U/mL and in newborns, from 4.3 to 110.7 U/mL. The mean AREase values for mothers were similar across three *PON1_192_* genotypes (Q/Q = 151.9 U/mL; Q/R = 144.3 U/L; R/R = 152.2 U/L; *p* = 0.64). In cord samples, the *Q192R* polymorphism slightly influenced AREase levels with *PON1_R192_* individuals having the highest average levels (Q/Q = 30.8 U/mL; Q/R = 35.8 U/mL; R/R = 42.9 U/mL), although the difference between genotypes was not statistically significant (*p* = 0.13).

AREase levels varied noticeably across *C-108T* genotypes in mothers (C/C = 163.6 U/mL; C/T = 147.1 U/mL; T/T = 134.8 U/mL; *p* = 0.04) with a larger gradient in newborns (C/C = 48.7 U/mL; C/T = 34.0 U/mL; T/T = 27.3 U/mL; *p* = 0.0003). AREase levels also differed by the *L55M* polymorphism in cord blood (*p* < 0.0001) but not in maternal blood (*p* = 0.44), with M/M homozygotes having the lowest levels in children (17.6 U/mL) and in mothers (135.6 U/mL). AREase levels were significantly higher in subjects with the *G-909* and *A-162* alleles in both mothers and children (*p* = 0.0003–0.03).

Mean POase and CPOase activities in mothers were significantly higher (1024.2 and 9358.3 U/L, respectively) than in newborns (315.1 and 2663.4 U/L, respectively) (Tables 4, 5). Both POase and CPOase activity levels demonstrated a strong association with the *PON1_192_* genotype. The lowest mean average POase activity was seen in *192_QQ_* children (81.2 U/L) and the highest in *192_RR_* mothers (1927.9 U/L), resulting in a 24-fold difference among these two genotype/age groups. Furthermore, the lowest overall POase level (10.3 U/L) was observed in the *192_QQ_* newborn child, and the highest in *192_RR_* adult (3014.2 U/L), bringing the overall difference among individuals from the same population to 300-fold. For CPOase, respective differences in enzyme activity between the lowest and highest levels in this cohort reached 70-fold. Both POase and CPOase activities also varied significantly by the other four *PON1* polymorphisms, with the lowest values for −909_GG_, −162_GG_, −108_TT_, and 55_MM_ homozygote groups (*p* < 0.001 in children; *p* < 0.03 in mothers).

Overall, PON1 activity levels in maternal blood were significantly higher than in cord blood for all enzyme assays and all genotypes (*p* < 0.001). Specifically, maternal POase, CPOase, and AREase levels were 3.3-fold, 3.6-fold, and 4.0-fold higher, respectively, than those in newborn. As expected, AREase, CPOase, and POase levels correlated well within *PON1_192_* genotypes. In mothers, the correlations between AREase and CPOase levels ranged between 0.62 and 0.74 in *QQ*, *QR*, and *RR* groups, and in newborns, these correlations were even stronger, 0.90–0.92 (all *p*-values < 0.0001 for both mothers and newborns). The correlations between AREase and POase, and between POase and CPOase were the highest in *RR* newborns (both 0.93) and mothers (0.7 and 0.95, respectively), and somewhat lower for other maternal and new-born *PON1_192_* genotype groups (all *p*-values less than 0.001). AREase activity was not compared with either CPOase or POase across genotypes because of the differential effects of the *PON1_192_* polymorphism on CPOase or POase activities. For example, the *PON1_Q192_* alloform hydrolyzes paraoxon with a catalytic efficiency nine times lower than *PON1_R192_* (Li et al. 2000).

### *Phenotypic effects of* PON1 *genotype and haplotype*

We constructed linear regression models to determine the proportion of the variance of AREase explained by the five *PON1* polymorphisms and the imputed haplotypes. The five *PON1* genotype polymorphisms explained 8.1 and 23.1% of the variance of AREase in mothers and newborns, respectively. The coefficient of variation (*R*^2^) was similar for both promoter polymorphisms *PON1*_−_*_909_* and *PON1*_−_*_162_* in mothers (~5%) and children (~14%) after adjusting for *PON1*_−_*_108_*. *PON1* haplotypes did not significantly improve the amount of variance explained (total *R*^2^ = 8.8% for mothers, 26.3% for newborns). The genetic contribution to AREase levels was significantly higher in newborns than in their mothers (*p* < 0.01). Because POase and CPOase characterize PON1 catalytic efficiency and are primarily controlled by *PON1_192_* polymorphism, a comparison of haplotype and genotype effects by these two assays was not relevant.

## Discussion

To our knowledge, this is the first study to report three PON1 enzyme activity levels in a large cohort of newborns and mothers from an agricultural cohort with relatively high levels of OP exposure. Two main PON1 factors are likely to contribute to the risk of adverse health effects of OP exposure: the level of enzyme (as measured by AREase assay) and the ability of this enzyme to detoxify OP metabolites (as measured in this study by CPOase assay, and primarily affected by *PON1_192_*). Thus, newborn children in this cohort, based on their lower PON1 plasma levels and detoxifying activities, are likely to be significantly more susceptible to OP exposure than are their mothers. Similar to results of a study from Mexico (Rojas-Garcia et al. 2005), we found large interindividual variability in PON1 plasma levels in both mothers and children, with a 14-fold difference in AREase among mothers, a 25-fold difference in newborns, and an overall range of 65-fold in this cohort. Additionally, we observed a range of 70-fold for CPOase and 300-fold for POase. However, it is important to emphasize that POase variability does not reflect differential sensitivity to paraoxon exposure based on recent animal data (Li et al. 2000). On the other hand, PON1 levels and *PON1_192_* polymorphism are very important in determining sensitivity to chlorpyrifos and chlorpyrifos oxon exposure (Cole et al. 2005; Furlong et al. 2006). Further, AREase variability primarily defines sensitivity to diazoxon exposure (Furlong et al. 2006; Li et al. 2000). Moreover, given the wide range in enzyme levels, some of the mothers are predicted to have an elevated susceptibility because they have levels as low as most of the newborns.

In nine children followed longitudinally, PON1 reached plateaus comparable with mean adult levels between 6 and 24 months of age (Cole et al. 2003). Our finding that CHAMACOS mothers had approximately 4-fold higher AREase levels than did their newborns confirms previous observations of lower PON1 activities in small groups of neonates compared with adults (Augustinsson and Barr 1963; Ecobichon and Stephens 1973; Mueller et al. 1983). Our finding is also consistent with a recent report where neonates had 2.6- to 4.6-fold lower PON1 levels compared with mothers in three ethnic groups residing in New York (Chen et al. 2003). However, no previous study has reported either POase or CPOase variability in such a large cohort of newborns.

In Latino newborns of the CHAMACOS cohort, all three *PON1* promoter polymorphisms as well as *PON1_55_* were significantly associated with AREase levels in children, and a greater proportion of the variance in AREase enzyme levels was explained by genetic polymorphisms in newborns than in mothers. The association of these polymorphisms and AREase levels is in agreement with another study of PON1 levels in newborns (Chen et al. 2005). There was a nearly complete LD among the three promoter region polymorphisms, as also observed in other studies (Brophy et al. 2001a; Chen et al. 2005; James et al. 2000; Rojas-Garcia et al. 2005). However, *PON1_192_* was not in LD with promoter SNP *PON1*_−_*_162_* and was in weak LD with the *PON1*_−_*_108_* and *PON1*_−_*_909_*. The lack of strong LD between *PON1_192_* and promoter polymorphisms is also in agreement with data from the Hispanic population in New York City (Chen et al. 2005). We found stronger LD between the two coding-region SNPs, *PON1_192_* and *PON1_55_*, in both mothers and children (*D*′ = 0.88 and 0.94, respectively) of the CHAMACOS cohort compared with Hispanics in New York City (Chen et al. 2005). The differences in linkage pattern may be attributed to variation among ethnic groups (Koda et al. 2004).

It has been reported that the association of the *M55* allele with low PON1 levels is primarily attributable to LD with the inefficient *T-108* allele (Brophy et al. 2001b). *PON1_M55_* has also been reported to be somewhat less stable than *PON1_L55_*, additionally affecting protein levels in plasma (James et al. 2000). This may explain why in CHAMACOS mothers, who have about a 4-fold higher AREase levels than the newborns, the effect of *PON1_55_* was not statistically significant.

Our analysis of five *PON1* SNPs in a Latino population of Mexican descent living in California suggests that these SNPs may be located on separate haplotype blocks because we found nearly complete LD among the coding SNPs but not between *PON1_192_* and *PON1*_−_*_162_* The presence of several haplotypes blocks in the *PON1* gene has been previously reported for other ethnic groups (International HapMap Consortium 2003; Koda et al. 2004). This underscores the importance of further analysis of *PON1* genetic variability. The gene frequencies for specific alleles of *PON1* genes vary by ethnicity, implying different population susceptibility to pesticides (Costa et al. 2002). The frequency of *PON1_192_* alleles in our Latina cohort of Mexican descent (*Q* = 0.5) was similar to those observed in Caribbean Hispanic mothers and neonates in New York City (both *Q* = 0.5) (Chen et al. 2003). *PON1*_−_*_162_* frequencies were also comparable in these two populations (~ 0.8). However, the frequencies for the *PON1*_−_*_108_*, *PON1*_−_*_909_*, and *PON1_55_* were noticeably different between Latinos from California and New York. The *PON1_192_* frequency in Hispanics from Washington State (*Q* = 0.6) was slightly higher than in New York and California (Brophy et al. 2002). In previous studies, the allele frequencies for *PON1_192_* polymorphism in Caucasians was *Q* = 0.7, whereas for African Americans and other groups of African descent, the *PON1_192_* frequencies are reversed, *Q* = 0.3 (Brophy et al. 2002).

Allebrandt et al. (2002) have compared a combination of the *PON1_192_* and *PON_155_* allele frequencies across various ethnic groups. Using this approach, Mexican Latinos of the CHAMACOS cohort appear to be equally differentiated from Caucasians, Asians, and African Americans, which is consistent with their Native American background, whereas Caribbean Hispanics from New York (Chen et al. 2003) are closer to Africans and Caucasians (data not shown). This difference across ethnic groups corroborates genetic and historical information about these populations (Cavalli-Sforza et al. 1993).

An effect of both *PON1* genotype and haplotypes on PON1 phenotype as measured by AREase was stronger in CHAMACOS newborns. This is in agreement with another study (Chen et al. 2005; Wetmur et al. 2005) that evaluated the relationship of five *PON1* SNPs with enzyme activity in mothers and their newborns. It is also clear that polymorphisms characterized to date in the *PON1* gene account for only a portion of the variability in PON1 levels observed among individuals. Additional research needs to be carried out to identify other factors (e.g., trans-acting factors, other *PON1* polymorphisms including intronic and exonic splice enhancing sequences) that influence PON1 expression.

Individuals with low PON1 activity are hypothesized to be at higher risk for any adverse health effects of OP exposure. In the only study to date to directly examine this hypothesis, Berkowitz et al. (2004) reported that in residents of east Harlem (the same cohort described by Chen et al. 2003), low PON1 plasma levels were associated with smaller neonatal head circumference. Further, although prenatal levels of the urinary metabolite of chlorpyrifos—3,5,6-trichloro-2-pyridinol (TCP)—were not associated with any measure of fetal growth or length of gestation by itself, higher levels of TCP were associated with smaller head circumference in children whose mothers had low expression of PON1.

We previously reported in the CHAMA-COS cohort that OP exposure as measured by urinary dialkyl phosphate metabolite levels of the mother during pregnancy was associated with shorter gestational duration (Eskenazi et al. 2004) and poorer neonatal reflexes (Young et al. 2005). A recent publication links *PON1_RR_* and *PON2_CC_* genotypes in infants with increased risk of preterm delivery in China (Chen et al. 2004). In future analyses, we aim to expand the analyses of PON1 to the entire CHAMACOS cohort and to determine whether PON1 levels modify the previously observed relationship between OP exposure and gestational duration and neonatal development.

## Figures and Tables

**Table 1:** *PON1* allelic frequencies in Latino mothers and their newborns.

Position in *PON1*	SNP	Mother (*n* = 130)	Children (*n* = 130)	Total (*n* = 260)
−*909*	C	0.48	0.44	0.46
	G	0.52	0.56	0.54
−*162*	A	0.21	0.19	0.20
	G	0.79	0.81	0.80
−*108*	C	0.51	0.55	0.53
	T	0.49	0.45	0.47
*55*	L	0.82	0.82	0.82
	M	0.18	0.18	0.18
*192*	Q	0.46	0.51	0.48
	R	0.54	0.49	0.52

**Table 2:** Tests of *PON1* LD in Latino mothers and children.

	*192*			*55*			−*162*			−*909*		
Position in *PON1*	*D*′	χ^2^	*p*-Value	*D*′	χ^2^	*p*-Value	*D*′	χ2	*p*-Value	*D*′	χ^2^	*p*-value
Mothers												
−*108*	0.22	6.0	0.01	0.74	17.9	< 0.001	0.81	35.1	< 0.001	0.90	182.0	< 0.001
−*909*	0.19	4.7	0.03	0.75	19.5	< 0.001	1.00	57.9	< 0.001			
−*162*	< 0.01	0.0	0.94	1.00	7.1	0.01						
*55*	0.88	29.7	< 0.001									
*192*												
Children												
−*108*	0.27	6.9	0.007	0.60	12.9	< 0.001	0.70	18.4	< 0.001	0.95	174.8	< 0.001
−*909*	0.34	9.9	0.002	0.72	19.1	< 0.001	1.00	38.7	< 0.001			
−*162*	0.18	0.7	0.41	0.63	2.7	0.11						
*55*	0.94	35.7	< 0.001									
*192*												

**Table 3:** Haplotypes (%) of five *PON1* polymorphisms in 130 Latino mothers and their newborns.

Haplotype	Mothers	Children
A:T:G:C:T^a^	17.3	16.3
G:T:G:C:T	16.6	24.3
G:T:G:G:C	16.4	17.4
A:A:G:C:T	12.3	11.8
A:T:G:G:C	10.4	8.0
G:T:A:G:C	9.1	8.8
A:T:A:G:C	9.0	6.7
Group 1, total	91.1	93.3
A:A:G:G:C	1.9	1.9
G:T:G:C:C	1.8	0.9
G:T:A:G:T	1.0	0.5
G:A:G:G:C	1.0	0.1
A:T:A:G:T	0.9	0.9
A:A:A:G:C	0.7	1.2
A:T:G:G:T	0.5	—
Group 2, total	7.8	5.5
Other	1.1	1.2

a Polymorphisms in five loci of *PON1* gene are listed in the following order: *192*(*A/G*), *55*(*A/T*), −*162*(*A/G*), −*909*(*C/G*), −108(*C/T*).

**Table 4:** *PON1* polymorphism frequencies and enzyme activities in Latina mothers (*n* = 130).

		AREase (U/mL)		POase (U/L)		CPOase (U/L)	
Genotype	Percent	Mean (range)	*p*-Value	Mean (range)	*p*-Value	Mean (range)	*p*-Value
−*909*	100.0						
CC	25.4	131.7 (54.5–233.7)		773.9 (150.5–2538.8)		8104.5 (3975.8–15389.2)	
GC	46.1	149.6 (19.8–242.8)	0.03	981.8 (66.1–2866.0)	0.001	9522.0 (1661.7–17098.0)	0.001
GG	28.5	160.7 (78.9–281.4)		1324.1 (217.4–3014.2)		10492.6 (4535.9–16732.4)	
−*162*	100.0						
AA	5.4	172.8 (78.9–281.4)		1570.1 (397.3–2373.1)		10766.3 (6465.7–15723.8)	
AG	31.5	160.8 (82.9–261.9)	0.02	1102.4 (217.4–3014.2)	0.03	10162.8 (4535.9–17098.0)	0.03
GG	63.1	139.8 (19.8–239.3)		939.4 (66.1–2866.0)		8958.8 (1661.7–16203.2)	
−*108*	100.0						
CC	27.3	163.6 (78.9–281.4)		1388.7 (217.4–3014.2)		10762.1 (4535.9–16732.4)	
CT	46.9	147.1 (19.8–242.8)	0.04	982.8 (66.1–2866.0)	0.0003	9342.1 (1661.7–17098.0)	0.001
TT	25.8	134.8 (54.5–233.7)		768.3 (150.5–2538.8)		8283.2 (3975.8–15389.2)	
*55*	100.0						
LL	66.9	151.7 (54.5–281.4)		1181.4 (150.5–3014.2)		9982.5 (3975.8–17098.0)	
LM	29.2	141.6 (19.8–242.8)	0.44	770.1 (66.1–1638.6)	0.0001	8541.7 (1661.7–13798.5)	0.002
MM	3.9	135.6 (102.0–185.5)		250.3 (212.0–339.7)		6684.0 (5391.1–9653.5)	
*192*	100.0						
QQ	30.0	151.9 (19.8–237.5)		340.7 (66.1–571.1)		8848.4 (1661.7–15389.2)	
QR	46.9	144.3 (72.9–261.9)	0.64	1064.0 (565.4–2058.8)	< 0.0001	9346.5 (4992.0–17098.0)	0.03
RR	23.1	152.2 (78.9–281.4)		1927.9 (1114.9–3014.2)		10577.1 (6465.7–16203.2)	
All genotypes		149.2 (19.8–281.4)		1024.2 (66.1–3014.2)		9358.3 (1661.7–17098.0)	

**Table 5:** *PON1* polymorphism frequencies and enzyme activities in Latino newborns (*n* = 130).

		AREase (U/mL)		POase (U/L)		CPOase (U/L)	
Genotype	Percent	Mean (range)	*p*-Value	Mean (range)	*p*-Value	Mean (range)	*p*-Value
−*909*	100.0						
CC	14.0	24.2 (6.3–110.7)		222.8 (10.3–802.6)		1993.9 (245.7–7664.0)	
GC	60.4	34.1 (4.3–108.8)	0.0001	272.6 (34.2–1235.8)	0.0004	2452.3 (485.2–7004.4)	0.0002
GG	25.6	48.9 (8.2–104.2)		431.4 (66.1–1352.0)		3395.5 (1231.3–7669.4)	
−*162*	100.0						
AA	3.1	57.7 (43.2–90.8)		707.4 (314.6–1352.0)		4977.3 (2715.3–7669.4)	
AG	31.8	42.6 (8.2–104.2)	0.006	317.4 (49.0–812.4)	0.0004	2819.8 (714.7–4851.5)	0.0002
GG	65.1	32.3 (6.3–110.7)		281.2 (10.3–1235.8)		2412.8 (245.7–7664.0)	
−*108*	100.0						
CC	27.6	48.7 (8.2–104.2)		426.3 (66.1–1352.0)		3359.5 (1231.3–7669.4)	
CT	54.3	34.0 (5.9–108.8)	0.0003	283.8 (49.0–1235.8)	0.0003	2479.4 (988.4–7004.4)	0.0003
TT	18.1	27.3 (6.3–110.7)		214.9 (10.3–802.6)		2102.8 (245.7–7664.0)	
*55*	100.0						
LL	67.2	42.9 (5.9–110.7)		364.9 (60.4–1352.0)		3008.4 (714.7–7669.4)	
LM	28.9	23.5 (4.3–50.4)	< 0.0001	195.0 (34.2–454.9)	< 0.0001	1902.0 (485.2–4108.1)	< 0.0001
MM	3.9	17.6 (6.3–54.2)		92.0 (10.3–286.1)		1171.0 (245.7–2123.2)	
*192*	100.0						
QQ	20.0	30.8 (6.3–108.8)		81.2 (10.3–178.6)		2074.3 (245.7–4778.5)	
QR	55.1	35.8 (4.3–110.7)	0.13	292.3 (34.2–802.6)	< 0.0001	2587.8 (485.2–7664.0)	0.006
RR	23.9	42.9 (5.9–104.2)		543.3 (165.3–1352.0)		3214.6 (988.4–7669.4)	
All genotypes		36.2 (4.3–110.7)		315.1 (10.3–1352.0)		2663.4 (245.7–7669.4)	
